# The relationship between parks and recreation per capita spending and mortality from 1980 to 2010: A fixed effects model

**DOI:** 10.1016/j.pmedr.2019.100827

**Published:** 2019-02-08

**Authors:** J. Tom Mueller, So Young Park, Andrew J. Mowen

**Affiliations:** a Department of Agricultural Economics, Sociology, and Education, College of Agricultural Sciences, The Pennsylvania State University, Armsby Building, University Park, PA 16802, United States of America; b Department of Recreation, Park, and Tourism Management, College of Health and Human Development, The Pennsylvania State University, 801 Donald H. Ford Building, University Park, PA 16802, United States of America

**Keywords:** Recreation, Mortality, Preventive medicine, Parks, recreational, Population, County government, Health expenditures

## Abstract

Evidence concerning the link between park access, use, programming and health has continued to grow. However, government funding for parks and recreation is highly susceptible to the ebbs and flows of the national economy. Given this, the purpose of this study was to test the relationship between county area spending on parks and recreation operations and all-cause mortality in the United States from the years 1980–2010. Using data from 1980 to 2010 collected from the U.S. Census Bureau and the Institute for Health Metrics and Evaluation, we analyzed the relationship between per capita county area spending on parks and recreation and county-level all-cause age-standardized female, male, and overall mortality using county and year fixed effects as well as relevant time-variant controls. The study was conducted during 2017 and 2018. County area spending on parks and recreation was negatively associated with overall and female-specific mortality from 1980 to 2010. According to our models for female and overall all-cause age-standardized mortality, when holding all else equal, a hundred-dollar increase in 2010 dollars in per capita parks and recreation operational expenditures was associated with an average decrease in morality of 3.9 and 3.4 deaths per 100,000, respectively. Although not commonly viewed as a form of healthcare spending, increased government funding for parks and recreation services had a significant association with decreased county level mortality. Our results suggest higher levels of per capita spending on parks and recreation may lead to lower levels of mortality.

## Highlights

• Higher per-capita parks and recreation spending was associated with lower all-cause mortality.
• The relationship held for female and overall mortality, but not male.
• Increased parks and recreation spending was associated with decreased mortality.
• Increasing government spending on parks and recreation may improve public health.

## 1 Introduction

It is predicted that by the year 2025 the United States will spend 19.9% of its gross domestic product (GDP) on healthcare (Centers for Medicare and Medicaid Services, 2017). United States spending on healthcare has experienced a constant increase during the period of 1960–2016, even during the Great Recession where it increased by 4.5%, while the GDP declined by 2.1% (National Health Expenditure Historical Data, n.d.). Although the U.S. spends more than any other country on healthcare, it still falls behind other developed nations on key health metrics such as Type II diabetes, heart disease, and other chronic conditions (World Health Organization, 2016). Given this, there have been mounting concerns among researchers and the public regarding the possibly diminishing returns of increased spending on public health, as well as interest in other, possibly more effective ways, of improving health outcomes (Murphy and Topel, 2003). Given that continued direct investment in healthcare is not the only form of investment with public health related outcomes, our study focused on the efficacy of an indirect form of ‘healthcare’ spending—parks and recreation—and its influence on a common measure of overall public health, all-cause mortality, from 1980 to 2010 (Mowen et al., 2017; Thacker et al., 2006). Considering the large economic value attributed to even modest decreases in mortality, a robust understanding of the impacts of indirect forms of healthcare spending, such as parks and recreation, on mortality is necessary for informing future policy (Murphy and Topel, 2006).

While healthcare spending may have experienced a consistent increase in spending from 1960 to 2016, parks and recreation spending did not share the same fate, as it is far more subject to the ebbs and flows of the national economy than other health-related services. Local government spending on parks and recreation experienced declines through the 1980's, a gradual climb throughout the 1990's and 2000's, and a steep decline following the Great Recession (Crompton and Kaczynski, 2003; Pitas et al., 2017a). Although parks and recreation spending has begun to recover post-recession, the recovery has proceeded more slowly than other government services, and as of 2014 had not recovered to pre-recession levels (Pitas et al., 2017a). While spending on parks and recreation may have decreased, evidence of the positive public health impacts of parks and recreation services has continued to expand (Bedimo-Rung et al., 2005; Cohen et al., 2007; Pitas et al., 2017b; Rosenberger et al., 2005; Rosenberger et al., 2009; Hughey et al., 2016; Zarr et al., 2017; Cohen et al., 2015; Mullenbach et al., 2018; Mueller et al., 2018).

Although not all parks and recreation services focus on nature and biodiversity, many do, and exposure to both nature and biodiversity has significant implications for population health (Kuo, 2015). Research has suggested that nature and biodiversity promote health through improved air quality (Kuo, 2015; Ruokolainen et al., 2017), exposure to allergens (Ruokolainen et al., 2017; Sandifer et al., 2015), decreased stress (Shanahan et al., 2015; de Vries et al., 2013), increased social cohesion (de Vries et al., 2013), and a decrease in mental health issues (Cox et al., 2017). Beyond the general impacts of nature and biodiversity, parks and recreation services have also been linked to increased physical activity, improved self-rated health, and lower obesity (Bedimo-Rung et al., 2005; Cohen et al., 2007; Pitas et al., 2017b; Rosenberger et al., 2005; Rosenberger et al., 2009; Cohen et al., 2015; Mullenbach et al., 2018; Mueller et al., 2018). However, there has yet to be research directly linking increased park and recreation spending and mortality. Previous research has demonstrated a consistent and positive relationship between leisure time physical activity and all-cause mortality, with moderate and high levels of physical activity reducing mortality risk by upwards of 20 and 30% (Löllgen et al., 2009; Nocon et al., 2008). However, while park access and investment have been linked to increases in physical activity, which should in turn decrease all-cause mortality rates, no previous research has evaluated the direct relationship between park and recreation spending and all-cause mortality (Cohen et al., 2007; Cohen et al., 2015; Löllgen et al., 2009; Nocon et al., 2008). The all-cause mortality rate is particularly suitable as an outcome variable of overall public health in this analysis due to evidence that spending on healthcare manifests in mortality impacts more readily than other metrics such as life expectancy (Gallet and Doucouliagos, 2017).

Prior research concerning the relationship between increased government spending on parks and recreation and health outcomes has reported mixed results (Rosenberger et al., 2005; Humphreys and Ruseski, 2007). Those studies that have explored the relationship between spending on parks and recreation and public health outcomes have been limited in their scope either by scale or by time (Rosenberger et al., 2005; Humphreys and Ruseski, 2007). Therefore, we used a fixed effects model to increase our understanding of the relationship between county area government spending on parks and recreation and mortality in the United States from 1980 to 2010 by testing the hypothesis that increases in county area per capita spending on parks and recreation operations were associated with decreases in county-level all-cause mortality over the period of 1980–2010. The use of county and year fixed effects treated each county as its own control, thus controlling for time-invariant confounding variables. By combining this approach with relevant time-variant controls, we estimated robust models evaluating the association between local government spending on parks and recreation and all-cause mortality.

## 2 Methods

### 2.1 Data sources

The data for our analysis were collected from four sources for the years of 1980, 1990, 2000, and 2010: the State and Local Government Finance Survey (SLGFS), the Decennial U.S. Census, the American Community Survey (ACS), and the age-standardized county level mortality rate estimates provided by the Institute for Health Metrics and Evaluation (IHME) (Historic County Area Finance Data, 2011; 2007 County Area Finance Data, 2018; 2012 County Area Finance Data, 2018; Manson et al., 2017; Institute for Health Metrics and Evaluation (IHME), 2016). Data were extracted and analyzed during 2017 and 2018.

The SLGFS is a census of government revenue and expenditures throughout the United States from the federal to the local level. A full census of governments is conducted in years ending in 2 and 7 and data are reported in a variety of categories, one of which is parks and recreation. We used data aggregated at the county area level. This means expenditures for a given category represent the amount spent on that category by the county government as well as all smaller governments within that county (U.S. Census Bureau, 2006). The use of county area, as opposed to simply county government, provided us with a more realistic picture of total government spending in an area, while also facilitating the inclusion of non-standard counties such as the Virginia consolidated cities and the New Orleans parishes. We assumed a lagged impact of parks and recreation spending and used data from the years ending in 7 to predict mortality in the decennial years. To improve interpretation of results, we adjusted the SLGFS data for inflation and converted each year of data into 2010 dollars, the final year of the study period. This was performed using the Bureau of Labor Statistics Consumer Price Index Inflation Calculator (Bureau of Labor Statistics Consumer Price Index Inflation Calculator, n.d.).

Demographic information for each county was collected from either the Decennial U.S. Census or the ACS. For the years of 1980, 1990, and 2000, all data were collected from the Decennial Census. Due to the U.S. Census Bureau's transition away from the long-form census in 2010, our 2010 demographic data came from both the U.S. Census and the 2008–2012 ACS estimates. All demographic data were extracted using the National Historic Geographic Information System from the Integrated Public Use Microdata Series (NHGIS-IPUMS) (Manson et al., 2017).

We extracted mortality rates for each county for 1980, 1990, 2000, and 2010 from datasets provided by the IHME for 1980–2014. We used age-adjusted all-cause mortality rate estimates for males, females, and overall. The county level mortality estimates were generated by the researchers from the IHME using data from the National Vital Statistics System and small area estimation (Manson et al., 2017; Dwyer-Lindgren et al., 2016). We merged the three datasets into time-consistent geographic units using the same protocol used by the IHME to generate their mortality estimates, wherein counties that changed boundaries over the study period were merged into larger time-consistent geographic areas (Dwyer-Lindgren et al., 2016).

### 2.2 Variables

To measure the association between investment in parks and recreation and mortality, we used the current operational expenditures on parks and recreation at the county area level from the SLGFS. Parks and Recreation is defined by the SLGFS as, “Provision and support of recreational and cultural-scientific facilities maintained for the benefit of residents and visitors.” (U.S. Census Bureau, 2006) Included within the parks and recreation SLGFS category are, “…golf courses, playgrounds, tennis courts, public beaches, swimming pools, playing fields, parks, camping areas, recreational piers and marinas, etc.; galleries, museums, zoos, and botanical gardens; auditoriums, stadiums, recreational centers, convention centers, and exhibition halls; community music, drama, and celebrations including public support of cultural activities.” (U.S. Census Bureau, 2006) More detailed spending categories were not available. Parks and recreation operational expenditures were in hundred USD and divided by population so that it was adjusted to the size of each county. We chose to use operational spending, as opposed to capital investment for two reasons. First, because operational spending includes employee compensation, contractual services, repair, and maintenance, operational spending is more consistent over-time and in many ways can represent the overall investment of a county area in their parks and recreation system. Second, parks and recreation programming is captured under operational spending, which has been shown to be associated with self-rated health, a strong correlate of mortality (Pitas et al., 2017b).

The outcome variable, all-cause mortality, was the age-standardized estimate of the annual rate of all-cause mortality within a county population. This rate is presented in terms of deaths per 100,000 people. It accounted for all causes of death and was extracted for male, female, and overall. We examined the relationship between the investment in parks and recreation and all three mortality rates.

As our fixed effects model does not control for time-variant confounding variables, we controlled for time-variant variables that would be expected to impact the mortality rate. First, lagged operational expenditures for health, hospital, and welfare per capita were included. Second, percent of population unemployed and a prosperity index—a weighted sum of percentage of population with bachelor's degree or above and median income—were added as confounding variables as wealth and education are critical factors for health and medical care (Mcgranahan and Songsore, 1994; Winkleby et al., 1990). Principal component analysis was used to take into account the correlations between the percentage of population with bachelor's degree or above and the median income so that the model did not suffer from multi-collinearity. Each was given a weight based on its contribution to the principal component to create the prosperity index, which was standardized with a mean of zero and standard deviation of one. The squared terms of the unemployment and prosperity index were included in the regression as we observed quadratic relationships with mortality. Third, as there can be underlying differences according to race and ethnicity, we included ratios of three race and ethnicity groups relative to total county population: non-Hispanic White, non-Hispanic Black, and Hispanic (Fiscella et al., 2002; Riolo et al., 2005; Gold et al., 2006; Kimmel et al., 2016). Fourth, we included three dummy variables for year with 1980 as the reference year—1990, 2000, and 2010—to account for decreases in mortality over time. Last, we included county level fixed effects to control for omitted variables and unique county effects. The use of county level fixed effects controlled for time-invariant omitted variables by exclusively looking at intra-county variation. Therefore, we analyzed, on average, how a county area's changes in per capita parks and recreation spending were associated with the same county area's changes in all-cause mortality from 1980 to 2010, while controlling for necessary time-variant confounding variables. Descriptive data of variables included in the models are presented in Table 1.

**Table 1:** Variables included in regression analysis of all-cause mortality in the United States from 1980 to 2010 with summary statistics.

Variable	M (SD)	Range
Age-Standardized All-Cause Mortality Rate (deaths per 100,000)		
Female	798.5 (121.2)	302.7 to 2293.9
Male	1256.6 (232.6)	375.7 to 3076.5
Overall	987.9 (156.7)	343.4 to 2542.9
Population unemployed, %	3.1 (1.4)	0 to 25.5
Prosperity Index^a^	0 (1.0)	−2.1 to 7.6
Current Operational Spending Per Capita^b^ (hundred USD)		
Parks and recreation	0.44 (0.6)	0 to 16.0
Health	0.64 (1.0)	0 to 2.95
Hospital	2.44 (1.3)	0 to 9.98
Welfare	0.67 (4.9)	0 to 4.17
Race and Ethnicity		
White population, %	86.0 (15.8)	2.9 to 100
Black population, %	8.6 (14.3)	0 to 86.5
Hispanic population, %	5.6 (11.8)	0 to 97.1

Note: Data analysis was conducted in 2017 and 2018.

a Prosperity Index is a weighted sum of percentage of population with bachelor's degree or above and median income standardized with a mean of zero.

b All per capita spending has been adjusted to 2010 dollars.

### 2.3 Statistical analysis

In total, the dataset included 3063 counties for four time points—1980, 1990, 2000, and 2010—and had 36,684 observations. We excluded all counties in Alaska due to data limitations, and we merged multiple counties in nine states to ensure historically stable units of analysis (Manson et al., 2017). Adams County, CO and New York City, NY were excluded to ensure robust and conservative results as they were outliers regarding mortality. The findings were stronger with the outliers included. The observations were divided into three separate datasets, each with 12,228 observations, according to the three types of all-cause mortality: female, male, and overall.

We conducted our analysis using panel linear fixed effects models with robust estimators of variance. The current operational expenditure variables were lagged, as we assume that the impact from the increased expenditures on mortality manifests after a lapse of time. For instance, 1997 expenditure data was used to predict 2000 mortality. We included both county and year fixed effects in the model. In total, three models were estimated, one for the female mortality rate, one for the male, and one for the overall. The analysis was conducted in Stata/MP, version 14.0 and a significance threshold of *p* < .05 was used. The xtreg command with robust estimator of variance, vce(r), and fixed effects option, fe, was used for the analysis.

To assure a robust model, we assessed multi-collinearity among the independent variables and performed sensitivity tests. We regressed the model with the non-lagged expenditure variables—meaning we averaged the data for the year ending in 7 preceding each decennial year and the year ending in 2 following each decennial year—and found the results to be consistent. We also conducted sensitivity tests with parks and recreation current operational spending as percentage of the total spending, and found it was not significantly associated with mortality. This indicates that the amount of investment in parks and recreation relative to the population size may be more important than spending relative to total government expenditures in the case of mortality.

## 3 Results

**Fig. 1:**
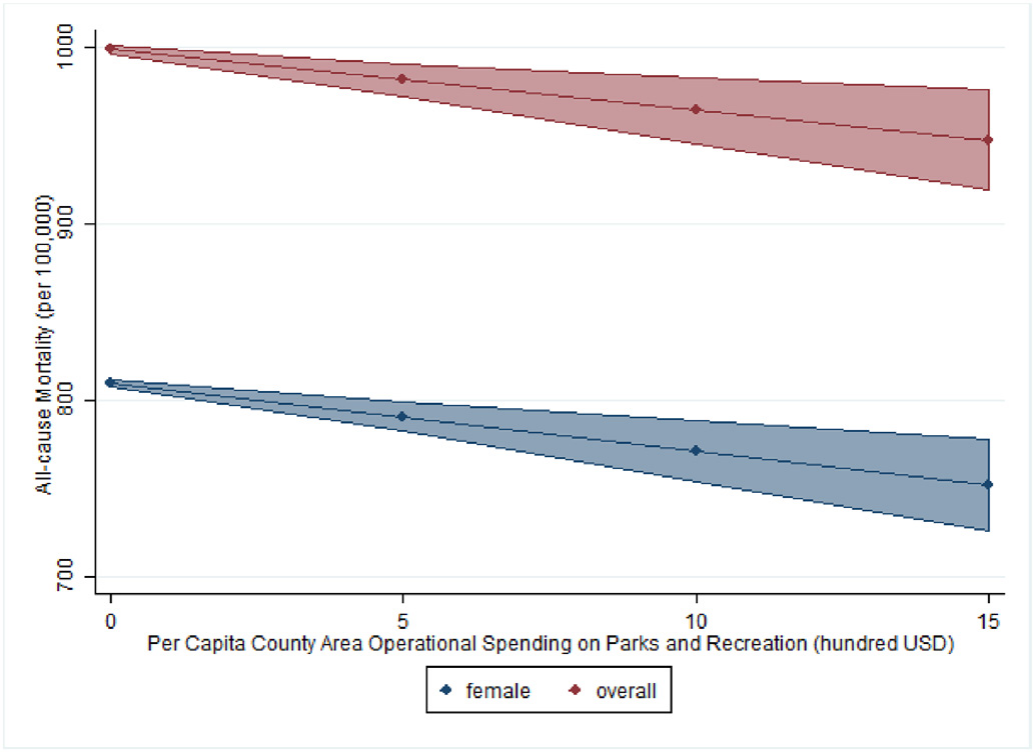
Estimated female and overall all-cause age-standardized mortality rates across the range of observed per capita parks and recreation operational expenditures (100 USD) from 1980 to 2010 while holding all other variables in the model at their mean.

All three models demonstrated strong effect sizes, with the model explaining 62% of the within county variation in the dependent variable for female mortality, 92% for male mortality, and 83% for overall mortality (Table 2). Our hypothesis was supported for female and overall mortality. We found operational expenditures on parks and recreation had a significant negative relationship with the mortality rate for female (*p* < .001) and overall mortality (*p* < .001). According to our models for female and overall all-cause age-standardized mortality, when holding all else equal, a one-hundred dollar increase, in 2010 dollars, in per capita parks and recreation operational expenditures was associated with a decrease in morality of 3.9 and 3.4 deaths per 100,000, respectively. Unlike the relationship with the female and overall mortality rate, the male mortality rate did not have a significant relationship with spending on parks and recreation at *p* < .05. To demonstrate the effect of spending on mortality, Fig. 1 presents the estimated value of overall and female mortality across the range of observed per capita spending levels while holding all other variables in the model at their mean.

**Table 2:** Fixed effects generalized linear regression results predicting all-cause age-standardized mortality rates (deaths per 100,000) in the United States 1980–2010.

Variable	Female		Male		Overall	
	Est. (SE)	*p*-value	Est. (SE)	*p*-value	Est. (SE)	*p*-value
Current operational spending per capita (3-year lag, hundred USD)						
Parks and recreation	**−3.9 (0.9)**	**<0.001**	−0.1 (1.2)	0.910	**−3.4 (1.0)**	**<0.001**
Health	**−2.4 (0.6)**	**<0.001**	**−3.2 (1.0)**	**0.001**	**−2.6 (0.7)**	**<0.001**
Welfare	**−4.0 (0.7)**	**<0.001**	**−3.6 (0.8)**	**<0.001**	**−4.0 (0.7)**	**<0.001**
Hospital	−1.6 (1.4)	0.262	0.0 (2.1)	0.985	−2.9 (1.5)	0.062

Demographics						
Prosperity Index	**−31.1 (0.8)**	**<0.001**	**−47.3 (3.9)**	**<0.001**	**−39.3 (3.0)**	**<0.001**
Prosperity Index squared	**−8.9 (0.8)**	**<0.001**	**−8.9 (1.1)**	**<0.001**	**−8.1 (0.9)**	**<0.001**
Population unemployed, %	**5.6 (1.5)**	**<0.001**	**6.8 (2.3)**	**0.003**	**7.0 (1.7)**	**<0.001**
Population unemployed squared	**−0.8 (0.2)**	**<0.001**	**−1.1 (0.3)**	**0.000**	**−1.0 (0.2)**	**<0.001**
White population, %	−0.3 (0.3)	0.330	0.3 (0.4)	0.423	0.1 (0.3)	0.846
Black population, %	0.1 (2.5)	0.849	**4.2 (0.7)**	**<0.001**	**1.5 (0.5)**	**0.003**
Hispanic population, %	−0.9 (0.3)	0.003	**−2.3 (0.4)**	**<0.001**	**−1.2 (0.3)**	**<0.001**
1980	[Reference]					
1990	**−26.4 (1.0)**	**<0.001**	**−109.5 (1.4)**	**<0.001**	**−59.7 (1.1)**	**<0.001**
2000	**8.4 (2.5)**	**0.001**	**−191.0 (3.5)**	**<0.001**	**−62.8 (2.7)**	**<0.001**
2010	**−53.8 (3.7)**	**<0.001**	**−336.5 (5.1)**	**<0.001**	**−154.1 (4.0)**	**<0.001**
Constant	**851.5 (29.0)**	**<0.001**	**1317.6 (36.6)**	**<0.001**	**1049.0 (30.2)**	**<0.001**
*R*^2^	0.6183		0.9155		0.8313	
*N*	12,228					

*Note*: Boldface indicates statistical significance (*p* < .05). Coefficients are from the fixed effects generalized linear regression model with control for year and county fixed effects, and robust estimator of variance. Regression coefficients represent the unit change in the age-standardized all-cause mortality rate (IHME, deaths per 100,000 population) as the link is linear. Within R^2^ is reported. All per capita expenditures have been adjusted to 2010 U.S. dollars. Data analysis was conducted in 2017 and 2018.

The included time-variant controls generally behaved as expected in our models. Among the other categories of current operational expenditures included in our model, health and welfare spending had significant and positive relationships with all three mortality rates (*p* < .001). The prosperity index that represented education level and income also showed a negative quadratic relationship. Percentage of population unemployed was positively correlated with all types of mortality, however the effect did taper off and become slightly negative when considering the quadratic term. For the race and ethnicity variables we found that the counties with a greater percentage of non-Hispanic Black population had higher mortality rates in the male and overall models.

## 4 Discussion

This study is the first, to our knowledge, linking government spending on parks and recreation to mortality using a robust fixed effects model. Our hypothesis, which predicted increases in county area spending on parks and recreation operations resulted in decreases in county-level all-cause mortality over the period of 1980–2010, was supported in two of the three models we tested. This indicates that as counties spent more per capita on parks and recreation operations, their female and overall mortality rates declined. While we are cautious to make claims of causality, the use of county and year fixed effects with time-variant controls in our models in many ways deals with the impact of omitted variables and provides compelling evidence of the direct link between parks and recreation government spending and mortality.

Previous research has linked park use, availability, and programming with measurable health outcomes (Bedimo-Rung et al., 2005; Cohen et al., 2007; Pitas et al., 2017b; Rosenberger et al., 2005; Rosenberger et al., 2009; Hughey et al., 2016; Zarr et al., 2017; Cohen et al., 2015; Mullenbach et al., 2018; Mueller et al., 2018). While a conceptual linkage between park funding, use, availability, programming and health could be made, our analysis provides robust empirical evidence linking funding and health. When considering the topic of healthcare spending, we view parks and recreation as an indirect form of healthcare spending. Evidence suggests that many individuals view parks and recreation as an essential component of the healthcare system (Mowen et al., 2017). Our results suggest this may be an accurate characterization. According to our results, government funded parks and recreation could certainly be argued to be an effective way to influence public health outcomes. In consideration of our findings, the instability of government funding for parks and recreation over the past 50 years is troubling (Pitas et al., 2017a). Given the emphasis in the public health community on preventive medicine, marked nationwide decreases in a dimension of spending that may have a significant impact on overall health is a cause for concern. While we found a direct relationship between spending and mortality, it is important to note that our analysis did not assess the specific pathways in which funding affects mortality rates. It is likely that decreases in mortality were achieved from many causes including improvements to local nature and biodiversity (Kuo, 2015), decreased stress (Shanahan et al., 2015; de Vries et al., 2013), improved air quality (Kuo, 2015; Ruokolainen et al., 2017), increased park use (Bedimo-Rung et al., 2005; Cohen et al., 2007), higher levels of park based physical activity (Bedimo-Rung et al., 2005; Cohen et al., 2007; Cohen et al., 2015), increased participation in parks and recreation programming (Bedimo-Rung et al., 2005; Pitas et al., 2017b), and improved social cohesion within communities (de Vries et al., 2013).

The presence of a robust association between parks and recreation spending and all-cause mortality in the case of female-specific mortality, but not male-specific mortality warrants further consideration. In short, our findings suggest that the male all-cause mortality rate is inelastic to changes in operational parks and recreation spending, relative to the female mortality rate. This finding may appear to be at odds with a number of observational studies showing that men both use parks more often and engage in park based physical activity more frequently than women (Cohen et al., 2007; Evenson et al., 2016). This begs the question, if men use parks more, shouldn't we see a change in the male mortality rate first? Although intuitive, we do not believe this should necessarily be the case. First, although research has shown that men use parks more frequently, it may be that the male population will use parks regardless of increases to operational spending. Research has consistently shown that men feel safer in public settings (Snedker, 2015). Operational spending supports staff and other safety measures that may make women more comfortable using parks and recreation services, but may have little impact on male use. Additionally, parks and recreation operational spending includes health-promoting park and recreation programming (e.g. exercise classes, organized hikes, walking tours). It is possible that women are more likely to utilize this type of service and thus benefit at a higher level than the male population, with the demographic make-up of two recent studies suggesting this may be the case (Han et al., 2015; Kling, 2018).

Additionally, changes in the all-cause mortality rate, unlike many other indicators of population health, require changes in actual deaths. For a host of reasons, mortality rates vary between males and females, with females having lower population mortality rates and longer life expectancies (Danaei et al., 2009; Vaidya et al., 2012; Austad and Bartke, 2016). Thus, it is possible that with a more fluid outcome variable we may see an impact on men's health. In fact, recent research found this to be the case in the relationship between operation spending on parks and recreation and self-rated health (Mueller et al., 2018). Ultimately it is likely a combination of multiple reasons that the impact of increases to park and recreation operational spending on male all-cause mortality is not significant in our analysis.

## 5 Study limitations

Our analysis was ecological and only used county level data, therefore individual characteristics are not included within our model. Items such as length of residence in a county, individual socio-economic indicators, and individual behaviors are not included. Future research should explore the effect of government spending on parks and recreation on individual health outcomes. Additionally, as this was the first paper linking parks and recreation spending and mortality, we chose to model the impact of spending on all-cause mortality. Investigation into the relative impact of parks and recreation on cause-specific mortality rates such as Type II diabetes, cancer, heart disease, and others will create a greater understanding of the nuance between these relationships. Regarding our independent variables, we were not able to disaggregate parks and recreation spending beyond the broad classification provided by the SLGFS, while we do not view this limitation as severe, it will be important for future research to find ways to understand the impacts of different specific forms of park and recreation spending. Further, we only considered localized spending on parks and recreation, it would be valuable for future research to find a way to consider all forms of spending, at multiple levels of government and private, when considering these relationships. Lastly, the geographic scale of the county masks unobserved heterogeneity within counties, while this scale was appropriate for our research questions, future research should explore this relationship at higher levels of specificity.

## 6 Conclusions

The ability to visit and use parks and recreation areas has previously been shown to influence health outcomes (Bedimo-Rung et al., 2005; Cohen et al., 2007; Pitas et al., 2017b; Rosenberger et al., 2005; Rosenberger et al., 2009; Hughey et al., 2016; Zarr et al., 2017; Cohen et al., 2015; Mullenbach et al., 2018; Mueller et al., 2018). The effect of parks and recreation spending on all-cause mortality from 1980 to 2010 was positive for overall mortality and female-specific mortality rates. This linkage demonstrates the importance of government funded parks and recreation services. We have demonstrated that as county areas spent more per capita on parks and recreation, mortality decreased. The inverse of this relationship, wherein decreases in spending could lead to an increase in overall mortality is cause for concern for the public health community. Park and recreation funding is often decreased in times of economic hardship in a manner more severe than other community services, our model suggests that these decreases have significant impacts on public health (Pitas et al., 2017a). We have demonstrated—somewhat unsurprisingly—that increases in funding for parks and recreation were associated with decreases in overall mortality over the past 30 years. In a time where people are searching for solutions to public health challenges, funding local parks and recreation services appears a valid approach.
